# Use of Medical Services and Medicines Attributable to Diabetes in Sub-Saharan Africa

**DOI:** 10.1371/journal.pone.0106716

**Published:** 2014-09-12

**Authors:** Jonathan Betz Brown, Kaushik Ramaiya, Stéphane Besançon, Paul Rheeder, Clarisse Mapa Tassou, Jean-Claude Mbanya, Katarzyna Kissimova-Skarbek, Eva Wangechi Njenga, Eva Wangui Muchemi, Harrison Kiambuthi Wanjiru, Erin Schneider

**Affiliations:** 1 Kaiser Permanente Center for Health Research, Portland, Oregon, United States of America; 2 Shree Hindu Mandal Hospital, Dar es Salaam, Tanzania; 3 NGO Santé Diabète, Bamako, Mali; 4 School of Health Systems and Public Health, Faculty of Health Sciences, University of Pretoria, Pretoria, South Africa; 5 Health of Populations in Transition-Research Group, Faculty of Medicine and Biomedical Sciences, University of Yaoundé, Yaoundé, Cameroon; 6 Department of Internal Medicine and Specialties, Faculty of Medicine and Biomedical Sciences and Laboratory of Molecular and Metabolic Medicine, Biotechnology Center, University of Yaounde, Yaoundé, Cameroon; 7 Institute of Public Health, Jagiellonian University Medical College, Krakow, Poland; 8 International Diabetes Federation, Brussels, Belgium; 9 Kenya Diabetes management and Information Centre (DMI), Nairobi, Kenya; 10 Health Systems Management, World Health Organization (WHO), Nairobi, Kenya; 11 The MAX Foundation, Edmonds, Washington, United States of America; University of Milan, Italy

## Abstract

**Background:**

Although the large majority of persons with diabetes and other non-communicable diseases (NCDs) lives and dies in low- and middle-income countries, the prevention and treatment of diabetes and other NCDs is widely neglected in these areas. A contributing reason may be that, unlike the impacts of acute and communicable diseases, the demands on resources imposed by diabetes is not superficially obvious, and studies capable of detecting these impacts have not be done.

**Methods:**

To ascertain recent use of medical services and medicines and other information about the impact of ill-health, we in 2008–2009 conducted structured, personal interviews with 1,780 persons with diagnosed diabetes (DMs) and 1,770 matched comparison subjects (MCs) without diabetes in Cameroon, Mali, Tanzania and South Africa. We sampled DMs from diabetes registries and, in Cameroon and South Africa, from attendees at outpatient diabetes clinics. To recruit MCs, we asked subjects with diabetes to identify five persons living nearest to them who were of the same sex and approximate age. We estimated diabetes impact on medical services use by calculating ratios and differences between DMs and MCs, testing for statistical significance using two-stage multivariable hurdle models.

**Findings:**

DMs consumed 12.95 times more days of inpatient treatment, 7.54 times more outpatient visits, and 5.61 times more medications than MCs (all p<0.001). DMs used an estimated 3.44 inpatient days per person per year, made 10.72 outpatient visits per person per year (excluding traditional healers), and were taking an average of 2.49 prescribed medicines when interviewed.

**Conclusions:**

In Sub-Saharan Africa, the relative incremental use of medical care and medicines associated with diagnosed diabetes is much greater than in industrialized countries and in China. Published calculations of the health-system impact of diabetes in Africa are dramatic underestimates. Although non-communicable diseases like diabetes are commonly thought to be minor problems for health systems and patients in Africa, our data demonstrate the opposite.

## Background

Non-communicable diseases (NCDs) account for the majority of disability and premature death in nearly all of the world’s countries. [1] Diabetes mellitus (DM) is an NCD of particular interest because untreated DM causes a wide range of other NCDs, including stroke, heart attack, renal disease, neuropathy, peripheral artery disease, lower-limb amputation and visual impairment. In 2011, DM accounted for an estimated 4.6 million deaths worldwide, more than HIV and malaria combined, and consumed at least 465 billion current U.S. dollars (USD) for its treatment. [2], [3] In most of the world, type 2 diabetes, the overwhelmingly predominant form, occurs in people on average ten years sooner and at a lower body mass index (BMI) than in populations of European heritage, [4], [5] and is linked to family and individual histories of famine [6]–[8] as well as to genetic inheritance, increased life expectancy, current diet, physical inactivity, [9] stress, [10] and social inequality. [11] Three-quarters of persons with diabetes live in low- and middle-income countries (LMICs) [2].

Little information is available for LMICs on the availability, use of, cost, and quality of medical care for diabetes. A recently published study covering 12 regions in China reported that, after direct adjustment for age, sex, and urban/rural location, individuals with diabetes received 1.93 times more days of inpatient treatment, 2.40 times more outpatient visits, and 3.35 times more medications than people with normal glucose tolerance. [12] Expenditures for medical care, adjusted for age and sex, were 3.38 times higher among Chinese with diabetes than among Chinese with normal glucose tolerance [12].

Data regarding resource utilization for diabetes care in sub-Saharan Africa are particularly scarce and difficult to generalize, although they all indicate that the diabetes-caused use of medical services and medications is substantial. Chale et al. interviewed 464 patients with diabetes at Muhimbili Medical Center in Dar es Salaam, Tanzania in 1989–90 and found that insulin and oral medicines accounted for nearly all their costs of medical care, in part because the salaries of physicians and nurses at that time were extremely low. [13] Despite the low salaries, however, annual per-patient expenditures for diabetes treatment exceeded Tanzania’s 1989 national income per capita, exceeded per capita spending on health by nearly 20-fold, [14] and accounted for 30.8% of total expenditures by the hospitals outpatient department. [13] Among hospital-using urban patients in Cameroon in 2001, Nkegoum calculated that direct medical care expenditures to treat diabetes totaled USD 489 per patient per year. Hospital admissions accounted for 56% of expenditures, followed by glucose lowering medicines (33.5%), laboratory tests (5.5%), and consultation fees (4.5%). [15] The International Diabetes Management Practices study (IDMPS) recruited a stratified random sample of physicians experienced in insulin therapy from various continents including one Sub-Saharan African country, the Republic of South Africa, in 2006–2007. Mean annual general practitioner visits in South Africa were 3.6 (4.8) per patient, mean number of hospitalizations per annum was 0.3 (1.2) and the number of emergency room visits was 0.1 (0.5). Micro- and macro-vascular complications as well as level of glycaemic control were positively correlated to hospitalization [16].

To obtain more complete and generalizable data for Sub-Saharan Africa, including good estimates of the diabetes to non-diabetes expenditure ratio, R, we decided to estimate the impact, cost, and availability of medical care for diabetes using identical, comparator-controlled methods in several Sub-Saharan countries simultaneously. In this paper we report the estimates of the use of medical services and medicines caused by diabetes.

## Methods

### Ethics Statement

In Mali approval was given by the Faculty of Medicine and Pharmacy and Dentistry, Research Ethics Committee of the University of Bamako; in Tanzania this was given by the Medical Research Coordinating Committee of the Ministry of Health and in South Africa by the Research Ethics Committee, Faculty of Health Sciences at the University of Pretoria. Patients gave written informed consent and the research was conducted in accordance with the declaration of Helsinki.

### Site and Sample Selection

In 2008–2009, Study teams from four Sub-Saharan African countries–Cameroon, Mali, Tanzania, and South Africa–participated in the study. We agreed upon a target sample size of 500 cases and 500 controls per country to provide 90% power to detect a 5 percentage point difference in rates and proportions between cases and controls in each country. Principal investigators (PIs) selected sampling locations and sampling sources within their countries based on representativeness and feasibility. In Cameroon, cases were identified and recruited consecutively from outpatient diabetes clinics at three hospitals in Yaoundé, the country’s capital and largest city (specifically, Yaoundé Central Hospital, the national referral and teaching hospital, where 80% of cases were obtained, the District Hospital of Biyem-Assi, and the District Hospital of Cité Verte). In Mali, thanks to the previous creation of diabetes registries derived from diabetes-clinic records, the recruitment pool included all persons with diagnosed diabetes living in or near the country’s three largest cities: Bamako, the national capital, population 1.8 million, Sikasso, population 130,000, and Tombuctou, population 58,000. Cases were probability-sampled in Bamako and sampled exhaustively in Siskasso and Tombuctou. In Tanzania, cases were selected randomly from a diabetes registry created from health facility records in Temeke, population 800,000, one of the three local government areas within the capital province, Dar es Salaam. In South Africa, cases were identified consecutively from the waiting room of the diabetes clinic at Mamelodi Hospital serving Mamelodi, a predominantly black township east of Pretoria (Tshwane Metropolitan area).

Once cases were identified and agreed to participate, eachcase was asked to identify five persons of the same sex and approximate age living closest to them. We then contacted and recruited one control subject for each case using this information. Potential controls who said they had been diagnosed with diabetes were excluded.

### The Interview

The 25-page interview questionnaire (Appendix A) was developed initially in English, translated into French, and then back-translated into English to perfect the translation. The medical utilization questions analyzed in the present paper were adapted to fit African circumstances from previously validated survey items. [17]–[18] and tested in two languages, English and French. English versions were field-tested in Kenya and Tanzania and French versions were field-tested in Mali until a final questionnaire, identical in all respects except language, was accepted. Interviewers were medical professionals who were trained and performed practice interviews prior to entering the field. In Tanzania, interviewers orally translated the questionnaire into Kiswahili when subjects were not fluent in English.

The interview included questions about: the participant’s use of allopathic and traditional medical services over the previous 90 days; payments made at the time of service for medical care services; medicines currently in the participant’s possession; the use, price, and sources of these medicines; the subject’s receipt of essential diabetes-related medical care, the subject’s diabetes-self-care practices, and health-related quality of life. All interviews were conducted by physicians or field workers who had been trained in a central location in each country. Prior to starting the interview, interviewers confirmed and recorded each subject’s informed voluntary consent to participate. Most of the interviews were completed in 2008 and occurred in the subjects’ homes.

### Data Entry and Verification

Interview answers were recorded on a paper form (Appendix A) and subsequently entered electronically by each study team using one of two data entry programs, one web-based and one personal computer-based, that the coordinating center developed in collaboration with the national study teams. The reliability of both data entry programs was verified by entering the entirety of the Tanzanian data using both methods and comparing the results both across programs and against the hard-copy records. In addition, countries using the interactive program were given spreadsheet copies of their data, which they then compared and corrected as necessary against their hard-copy records. Finally, prior to analysis, the coordinating center scoured every variable in each country’s database for data that were out of range or inconsistent with other variables. Suspect values were then re-checked against the hard-copy records by the national study teams and excluded from the analysis dataset if a correct response could not be found in the hard-copy interview form. Prior to substantive data analysis, we reduced two extreme outlier values to the level of the next-largest unmodified value. Also prior to analysis, to prevent bias arising from rare and uncharacteristically long hospital stays, we Winsorized [19]–[20] the hospitalization data by reducing two extreme outlier values for hospital days to the level of the next-largest unmodified value in the dataset.

### Estimation of Annual Use of Medical Care

As described, the study team relied on participant recall to ascertain use of medical care services. To increase accuracy, we limited recall to a 90-day window and, to calibrate the window, asked respondents to name and associate a well-remembered event that had occurred approximately 90 days previously. To learn about the use of medicines, interviewers asked subjects to show them the medicines they were using and to tell them about their most recent purchase of each medicine. To further increase accuracy, and to control respondent burden, we asked for details only about the most recent hospital outpatient visit, overnight hospitalization, and purchase of each medicine. The study team then used the descriptions of this subset of most-recent events to estimate the characteristics of all events of the same kind, such as reasons for admissions and visits and mean length of hospital stay.

Annual rates of use of outpatient medical services were calculated by multiplying the amounts self-reported for the preceding 90 days by 4.0. [12] Although participants were asked about visits to hospital emergency wards, too few such visits were reported to support analysis, probably because emergency room visits were defined to exclude emergency-ward visits that resulted in an overnight stay. We excluded data on emergency-ward visits from this report. To estimate the last 90 days and annual number of medicines taken we used the self-reported number of pills per day and number of days per week these medicines were reported to be frequently taken. The recall window for hospitalisations was 365 days.

### Hypotheses and Hypothesis Testing

The study team’s *a priori* primary hypotheses were that total per-person hospital admissions, inpatient days, outpatient visits, and purchases of medicines by persons with DM would exceed total per-person use by matched controls (MCs), after adjustment for any residual differences in age and sex that were not directly controlled by matching. These hypotheses could not be tested using one-stage statistical procedures because most subjects had had no admissions during the last 90 days, a few had no outpatient visits, and some were taking no medicines when interviewed. Therefore, we used two-stage “hurdle” models [21] to test for differences in total service utilization. In the first stage, we used multivariable logistic regression models that included age, sex, and case status (DM = 1, MC = 0) to test for differences in the proportion of subjects with a non-zero value on the utilization event of interest, e.g., persons with at least one overnight admission or outpatient visit (OPV) during the preceding 90 days, or at least one current medicine (analysis done only among persons who reported use of at least one type of medical services). Then, we estimated a second-stage multivariable model using the same independent variables on a dataset containing only the observations with non-zero values. The functional form of the second-stage model depended on the underlying distribution of values: for counts of admissions, OPVs, and medicines, a Poisson model was used, while for total days in hospital, i.e., cumulative length of stay among having at least one hospital admission (if admitted), we checked for approximate normality and used ordinary least squares (OLS). Models were estimated using the free and open-source *R* program, which did not yield a single overall confidence interval for both stages simultaneously. However, if the coefficient on DM status is positive and significantly different from zero in the first model, and not significantly negative in the second model, then the two models together may be considered to confirm a utilization-increasing effect of diabetes. Because our primary hypotheses were one-sided, we used one-sided tests of significance. We considered but decided not to use random-effects models because our study locations were not intended to approximate a random sample of their respective countries or of Africa. For the same reason, we omitted site from our statistical models. (We evaluate the representativeness of our sites and subjects in the Discussion section).

### Tabulation of Healthcare Use

The absolute amount of medical care associated with, and arguably caused by diabetes can be estimated by subtracting results for matched comparison subjects from results for persons with DM. However, for comparison to other studies conducted at other places and times, a more robust statistic is the adjusted *ratio* of medical service use among persons with DM to the medical service use by MCs (DM:MC ratio). Ratios are much less influenced than absolute differences by variations in source data, recruitment methods, and differences in health systems and patterns of medical practice [3], [22]–[24].

## Results

Usable data were obtained on 1780 cases with diagnosed diabetes and 1770 matched controls. The mean age of DMs was 54.8 years; 64.7 percent of DMs were women. MCs were very similar to DMs, with mean age 53.7 and 65.0 percent women (Table 1). Subjects were older in South Africa (mean age 61.0 and 60.5 for DMs and MCs, respectively) and more likely to be female in Mali and South Africa. Although cases and comparison subjects were not directly matched on family income, we observed similar self-reported annual family incomes per capita, averaging USD 2570 for DMs and USD 2280 for comparators. Family income varied by country: it was lowest in Tanzania (DM: USD 1231) and highest in Cameroon (DM: USD 3917). In all countries, family size, defined as “the number of people who normally eat with you where you live,” was slightly larger among DMs than among MCs, 8.6 vs. 7.7. In Mali, family size was particularly large by our definition because extended families usually dine together in that country (15.7 for DMs and 13.9 for MCs). DMs and MCs were very similar in educational attainment, with 25.2% and 24.3% reporting no formal schooling, respectively, although highest level of schooling completed varied widely by country, with 47.0% and 26.6% of DMs reporting no formal schooling in Mali and Tanzania, respectively; by contrast, 14.4% of the DMs in Cameroon had attended university, compared to 1.0% or fewer in the other three countries.

**Table 1:** Characteristics of study samples by country and study site.

	N		Mean age		% Women		Mean annual familyincome per person (USD)		Family size	
Site	DM	MC	DM	MC	DM	MC	DM	MC	DM	MC
CAMEROON										
Yaoundé	497	499	56.14	55.16	52.11	51.90	3917	3067	6.79	5.58
MALI										
Bamako	297	301	51.57	49.59	81.00	81.76	2625	2247	15.56	13.37
Sikasso	150	148	55.05	54.79	62.16	62.67	2647	2697	19.06	17.45
Tombouctou	50	49	50.45	50.68	75.51	76.00	2734	2508	6.67	6.20
TOTAL	497	496	52.50	51.27	74.85	75.40	2636	2356	15.73	13.88
TANZANIA										
Temeke	485	474	51.82	50.46	57.73	58.23	1231	1137	5.48	5.12
SOUTH AFRICA										
Mamelodi	301	301	61.02	60.52	80.07	80.07	2383	2564	4.95	4.84
ALL SITES	1780	1770	54.77	53.71	64.69	65.02	2570	2280	8.62	7.66

DM means persons with diagnosed diabetes mellitus. MC means matched control group, persons without known diabetes of the same sex and approximate age, living near to the case with DM with whom they were matched. Mean annual family income per person is the respondent’s self-reported family income divided by the self-reported number of persons in the respondent’s family (family size).

### Use of Inpatient Care

As shown in Table 2, the rate of admissions per person was 0.049 among persons with DM during the preceding 90 days, compared to 0.011 among matched comparison subjects. After accounting for multiple admissions each person in the study group with diabetes averaged 0.28 admissions per person per year, compared to 0.045 admissions per year for MCs, a 6.63-fold difference. Because MCs reported so few hospital admissions, we were unable to tabulate age-group-specific differences or ratios in admission rates or duration of inpatient stay by age-group.

**Table 2:** Use of Inpatient Services.

	Sex		Age in Years					Years Since Diabetes Diagnosis				All
	Female	Male	<40	40–49	50–59	60–69	70+	<2	3 to 5	6 to 10	>10	
N (DM)	1152	628	178	398	556	436	212	477	508	476	291	1780
N (MC)	1150	620	213	408	583	388	178					1770
Percentage of Persons with At Least 1 Inpatient Admission, Last 90 Days												
DM	4.43	5.73	3.93	3.77	4.86	5.73	6.13	7.13	3.54	2.94	6.90	4.89
MC	1.39	0.49	1.00	0.96	0.51	1.99	1.52					1.07
Diff.	3.04	5.24	2.94	2.81	4.35	3.75	4.61					3.81
Ratio	3.19	11.67	3.94	3.93	9.53	2.89	4.02					4.55
Mean Number of Admissions if Admitted, Last 90 Days												
DM	1.63	1.18	1.00	1.08	1.50	1.24	0.80	1.28	1.34	1.66	1.05	1.42
MC	1.00	0.63	0.35	0.64	0.85	0.77	0.50					1.03
Diff.	0.63	0.56	0.65	0.44	0.65	0.47	0.30					0.39
Ratio	1.63	1.90	2.83	1.69	1.77	1.61	1.59					1.38
Mean Length of Stay, Most Recent Hospital Admission, if Admitted												
DM	10.08	14.74	7.38	10.73	19.64	12.41	6.39	12.91	11.16	14.47	10.18	12.15
Ratio	NA	NA	NA	NA	NA	NA	NA					2.07
Mean Annual Inpatient Admissions per Person, all Study Subjects												
DM	0.28	0.28	0.16	0.16	0.27	0.47	0.28	0.35	0.17	0.29	0.29	0.283
MC	0.06	0.03	0.04	0.04	0.02	0.09	0.06					0.045
Diff.	0.23	0.25	0.12	0.12	0.25	0.38	0.22					0.238
Ratio	5.12	10.71	3.94	4.19	13.06	5.08	4.64					6.27
Mean Annual Inpatient Days, per Person, all Study Subjects												
DM	2.87	4.13	1.16	1.72	5.23	5.81	1.81	4.55	1.85	4.26	2.95	3.44
MC												0.27
Diff.												3.17
Ratio												12.95

DM means persons with diagnosed diabetes mellitus. MC means age- and sex-matched comparison subjects. Data on Length of Stay for MCs are not available (NA) because sample size was insufficient to calculate. Diff. means DM−MC. Ratio means DM/MC. See text for description of statistical significance.

The first stage of the hurdle model confirmed that persons with DM were more likely to have had at least one admission than persons with MC after indirect multivariable adjustment for age and sex (p<0.01). In the second stage of the hurdle model, the mean number of admissions per person, if admitted during the preceding 90 days, was also higher in persons with DM (p<0.01) in a multivariable zero-truncated Poisson count model. Thus, the hypothesis that DMs experienced more admissions than MCs was confirmed.

Hospital stays were about twice as long for persons with DM (12.15 days) than for MCs (5.88 days, data not shown), so total annual inpatient days per person were 12.95 times higher among DMs (3.44 inpatient days per person per year for DMs versus 0.27 inpatient days per person per year for MCs). As presented in Table 2, among persons with DM in the study sample, annual inpatient admissions and especially annual inpatient days per person increased with age and formed a U-shaped pattern as a function of increasing self-reported time since diagnosis. The second stage of the hurdle process confirmed that total hospital days were higher for DMs than for MCs (p<0.01).

### Use of Outpatient Care

Table 3 displays the self-reported use of outpatient services by diabetes status, age, sex and four groups of diabetes duration. Over 90 percent of DMs (90.5%) reported having at least one outpatient in the last 90 days. The 90-day average for total visits, among DMs with at least one visit, was 2.8. By comparison, only 15.4 percent of MCs reported at least one visit during the previous 90 days and they reported 1.72 visits over the period if they had had at least one visit. Excluding visits to traditional healers, nearly all (10.3 out of 10.7) OPVs by DMs were to hospital-based clinicians (doctors or nurses). Relative to their use of allopathic OPVs, MCs used traditional healers frequently, 1.04 visits out of 2.73 total visits per year (38.1%). DMs averaged slightly more annual visits to traditional healers, 1.37, but traditional healers accounted for a much lower proportion of total DM OPVs (11.3% of 12.09 total visits per year).

**Table 3:** Use of Outpatient Services.

	Sex		Age in Years					Years Since Diabetes Diagnosis				All
	Female	Male	<40	40–49	50–59	60–69	70+	<2	3 to 5	6 to 10	>10	
Estimated Percentage of Persons with at least 1 Outpatient Visit, Last 90 Days												
DM	90.17	91.11	95.98	91.91	90.68	86.93	90.96	90.74	90.23	90.67	91.72	90.50
MC	17.95	10.80	11.72	14.03	14.32	21.21	14.20					15.44
Diff.	72.21	80.31	84.25	77.88	76.36	65.72	76.76					75.05
Ratio	5.02	8.44	8.19	6.55	6.33	4.10	6.41					5.86
Mean Outpatient Visits per Person, among those with at least 1 Outpatient Visit during the Last 90 Days												
DM	2.71	3.05	2.66	2.57	3.16	2.90	2.46	3.05	2.75	2.60	3.04	2.83
MC	1.73	1.92	1.33	1.50	2.10	1.63	1.91					1.78
Diff.	0.99	1.14	1.34	1.07	1.06	1.27	0.55					1.05
Ratio	1.57	1.59	2.01	1.71	1.50	1.78	1.29					1.59
Mean Annual Visits to Hospital Outpatient Departments, per Person (only among users or among all study subjects?)												
DM	9.92	10.99	10.07	9.44	11.49	10.30	9.03	11.03	9.96	9.52	11.34	10.30
MC												
Diff.												
Ratio	8.01	13.95	11.61	11.53	9.81	7.29	9.04					9.53
Mean Annual Visits to Private Doctors Outside of Hospital Clinics, per Person (only among users or among all study subjects?)												
DM	0.22	0.49	0.11	0.21	0.33	0.23	0.81	0.16	0.20	0.38	0.65	0.31
MC												
Diff.												
Ratio	0.77	2.74	2.82	1.86	1.46	0.69	1.30					1.27
Mean Annual Visits to Nurses Outside of Hospital Clinics, per Person (only among users or among all study subjects?)												
DM	0.12	0.09	0.09	0.25	0.09	0.01	0.10	0.12	0.09	0.13	0.08	0.11
MC												
Diff.												
Ratio	1.07	1.28	4.63	3.44	0.70	0.09	1.89					1.13
Mean Annual Visits to Traditional Healers, per Person (only among users or among all study subjects?)												
DM	1.37	1.38	1.42	0.83	1.50	1.97	1.03	1.94	0.57	1.86	1.15	1.37
MC												
Diff.												
Ratio	1.13	1.83	2.06	0.87	1.25	1.40	1.65					1.31
Mean Total Annual Outpatient Visits to a Any Clinician (Excluding Traditional Healers) (only among users or among all study subjects?)												
DM	10.25	11.57	10.27	9.91	11.91	10.54	9.93	11.31	10.25	10.03	12.07	10.72
MC	1.63	1.04	0.93	1.01	1.52	1.85	1.67					1.42
Diff.	8.62	10.53	9.35	8.90	10.39	8.69	8.26					9.30
Ratio	6.29	11.16	11.08	9.85	7.83	5.69	5.94					7.54

DM means persons with diagnosed diabetes mellitus. MC means age- and sex-matched comparison subjects. Diff. means DM−MC. Ratio means DM/MC. See text for description of statistical significance.

Overall, not counting visits to traditional healers, persons with diabetes reported 7.54 times more ambulatory medical encounters per year than matched subjects who did not have diabetes. Among DMs, visits per year did not vary significantly by age or by duration of diagnosed diabetes. Older MCs reported more OPVs per year than younger MCs.

With respect to the formal hypothesis that persons with DM used more OPVs than MCs, the coefficient on diabetes was statistically significant (p<0.01) in a positive direction in both the first-stage, zero hurdle logistic model and in the second-stage Poisson-regression count model, confirming the *a priori* hypothesis.

Our study-wide averages conceal considerable variation among the four countries. Total annual allopathic OPVs for DMs were much higher in Cameroon (13.41) and Mali (14.09) than in South Africa (5.54) and Tanzania (7.73), data not shown. DMs in Cameroon and especially in Mali were much more likely to use traditional healers than DMs in South Africa and Tanzania (Cameroon and Mali: 0.97 and 3.74 visits/year, respectively; South Africa and Tanzania: less than 0.01 and 0.049 visits/year, respectively).

Table 4 shows the causes of hospital admissions and hospital outpatient visits, as reported by the subjects. Half (49.6%) of the hospital admissions of DMs were attributed to “diabetes” rather than to any particular diabetic complication, such as heart disease (5.6%) or stroke (0.8%). MCs attributed 38.9% of their admissions to “other,” and recalled very few admissions for problems that can be caused by diabetes. DMs attributed fully 95.1% of their most recent hospital outpatient visits to “diabetes,” while MC attributed 73.0% of their visits to “other”.

**Table 4:** Self-reported causes of inpatient admissions and outpatient visits.

	Inpatient Admissions				Hospital Outpatient Visits			
	Diabetes		Matched Controls		Diabetes		Matched Controls	
Reason For Admission	N	%	N	%	N	%	N	%
Heart Disease	7	5.6	0	0.0	15	0.9	43	16.1
Stroke	1	0.8	1	5.6	0	0.0	2	0.7
Kidney Disease	0	0.0	0	0.0	4	0.2	2	0.7
Eye Disease	5	4.0	1	5.6	14	0.9	10	3.7
Leg or Foot Ulcer	3	2.4	0	0.0	1	0.1	4	1.5
Cancer	1	0.8	1	5.6	0	0.0	1	0.4
Lung Disease	6	4.8	1	5.6	1	0.1	4	1.5
Trauma	2	1.6	2	11.1	8	0.5	1	0.4
Diabetes	62	49.6	0	0.0	1541	95.1	0	0.0
Childbirth	1	0.8	2	11.1	0	0.0	5	1.9
Digestive Problems	7	5.6	2	11.1	0	0.0	0	0.0
Other	30	24.0	7	38.9	36	2.2	195	73.0
Does Not Know	0	0.0	1	5.6	0	0.0	0	0.0

### Use of Medicines

Table 5 summarizes the use of medicines by DMs and MCs. Nearly all cases with DM (94.8%) possessed and reported taking at least one medicine at the time they were interviewed, compared to one-fourth (26.7%) of matched controls. DMs who were using a medicine also used more medicines in total than MCs who were using a medicine (2.60 vs. 1.60 medicines per person). The mean number of medicines used per person with DM in the study (2.49) was 5.61 times greater than the mean number of medicines used by MCs (0.44). The DM vs. MC difference in use of any medicine was highly statistically significant (p<0.01) in the first-stage logistic model; in the second-stage Poisson model, the mean number of medicines among persons taking any medicine was also significantly higher for DMs (p<0.01). The combination of these two results confirms the study hypothesis for medicine use.

**Table 5:** Use of Medicines.

	Sex		Age in Years					Years Since Diabetes Diagnosis				All
	Female	Male	<40	40–49	50–59	60–69	70+	<2	3 to 5	6 to 10	>10	
N (DM)												
N (MCG)												
Percentage of Persons Taking at Least 1 Medicine												
DM	95.57	93.31	91.57	94.22	94.24	96.10	97.17	90.15	95.08	96.85	98.26	94.78
MC	30.63	19.33	13.34	17.42	29.85	32.41	39.13					26.66
Diff.	64.94	73.98	78.23	76.80	64.40	63.69	58.04					68.12
Ratio	3.12	4.83	6.86	5.41	3.16	2.96	2.48					3.56
Mean Number of Medicines Taken, if Taking Any												
DM	2.71	2.42	1.66	2.18	2.56	3.01	3.44	2.23	2.50	2.66	3.10	2.60
MC	1.66	1.47	1.06	1.30	1.53	1.79	1.96					1.60
Diff.	1.05	0.95	0.59	0.88	1.03	1.22	1.47					1.00
Ratio	1.63	1.64	1.56	1.68	1.67	1.68	1.75					1.62
Mean Annual Number of Medicines Taken per Person (among all Study Subjects)												
DM	2.61	2.28	1.52	2.07	2.44	2.92	3.36	2.02	2.40	2.59	3.06	2.49
MC	0.53	0.28	0.16	0.24	0.45	0.61	0.81					0.44
Diff.	2.07	2.00	1.37	1.83	1.99	2.31	2.55					2.05
Ratio	4.88	8.18	9.73	8.62	5.46	4.79	4.13					5.61

MC means age- and sex-matched comparison subjects. Diff. means DM−MC. Ratio means DM/MC. In this table, medicines are defined to exclude herbs and other traditional medicines. See text for description of statistical significance.

Unlike annual outpatient visits per person, medicines used per person increased with age and with the length of time since diagnosis among DMs (both p<0.01) and, for MCs, increased with age (p<0.05). As presented in Table 6, except in Tanzania, DMs purchased three-fifths to three-quarters of their medicines from public hospital pharmacies. In Tanzania, DMs bought 84.4% of medicines from private pharmacies.

**Table 6:** Sources of medicines (%).

	Private Pharmacy	Hospital Pharmacy
Site		
CAMEROON		
Yaoundé	42.2	57.8
MALI		
Bamako	35.6	64.4
Sikasso	42.3	57.7
Tombouctou	26.3	73.7
All Sites	36.7	63.3
TANZANIA		
Temeke (Dar es Salaam)	84.4	16.6
SOUTH AFRICA		
Mamelodi (Tshwane)	25.0	74.5

## Discussion

We interviewed samples of persons with diagnosed diabetes and age-, sex-, and residence-matched comparison subjects without diagnosed diabetes in Cameroon, Mali, South Africa and Tanzania. We found that the use of medical services and medicines was dramatically higher among DMs than among MCs. DMs used 6.27 times more inpatient admissions, 12.95 times more days of inpatient treatment, 7.54 times more outpatient visits, and 5.61 times more prescription medications. DMs used an estimated 3.44 inpatient days per person per year, made 10.72 outpatient visits per person per year (excluding traditional healers), and were taking a average of 2.49 prescribed medicines when interviewed. These ratios are much higher than recently published ratios for similar comparisons in developed countries (average 2.0–2.4 for total medical expenditures) [25]–[26] and much higher than the ratios observed in a companion study using a similar interview schedule and case-control design in China, where the medical services use ratios of DMs relative to subjects with normal glucose tolerance (NGT) were 1.93 for inpatient days, 2.40 for outpatient visits, and 3.35 for medicines [12].

A number of factors combine to explain why persons with diagnosed diabetes in our study used so many more medical services. One important factor is that, unlike much of the industrialized world in recent years, and unlike our companion study in China that ascertained diabetes by population-based household screening, a majority of diabetes cases in Africa remain undiagnosed. [27] In Africa, testing for diabetes usually occurs after patients present with complications known to arise from diabetes. [27] This means that, as a group, persons recognized as having diabetes in Africa (and in other LMICs) are much sicker than their counterparts in many other places, and need more services and medications.

A second major cause of the high relative cost of diabetes care in Africa–and another reasons why Africans diagnosed with diabetes are much sicker–is that Africans with diabetes are not given inexpensive basic medicines to control hypertension and dyslipidemia, and also probably do not have good blood sugar control. [14] This results in expensive, disabling complications, [28]–[32] which greatly increase the need for medical services [33]–[35].

In this connection, we note that in other data from our study, a high portion of medical care costs in Africa are incurred by patients, not covered by insurance, government or charity. As shown in Table 7, to pay their medical expenses many families must use their savings, borrow from friends and relatives, or sell their assets. DMs were over four times more likely than MCs to report selling possessions, houses or land to raise funds to pay for medical care (Table 7). As a result, people with diabetes may not seek required preventive care, increasing the risk of complications, which are more cost yet (if treated).

**Table 7:** Self-reported sources of funds to pay for medical care (%).

	Cameroon		Mali		South Africa		Tanzania		All Sites		
	DM	MC	DM	MC	DM	MC	DM	MC	DM	MC	DM/MC
Current household income	80.7	67.7	61.4	43.3	70.4	69.1	86.4	90.3	75.1	67.2	1.1
Social welfare support	3.4	0.8	36.2	19.8	67.1	55.0	12.6	9.9	25.8	17.8	1.5
Donations by employers or agencies	0.8	1.4	3.0	1.8	0.0	0.0	0.8	0.2	1.3	1.0	1.3
Nearby friends or family	51.5	36.3	9.7	8.5	6.6	7.0	34.4	22.2	27.6	19.7	1.4
Family living abroad	11.9	5.2	27.8	17.1	1.3	1.7	1.2	0.8	11.6	6.8	1.7
Savings	28.2	28.3	4.4	5.0	16.3	14.3	6.0	9.1	13.5	14.3	0.9
Borrowing	40.4	24.6	5.4	1.0	19.3	4.7	10.7	13.5	19.0	11.6	1.6
Selling possessions	6.0	1.0	5.6	1.6	0.0	0.0	0.2	0.2	3.3	0.8	4.2
Selling housing or land	9.3	2.4	8.5	1.6	0.3	0.3	0.6	0.2	5.2	1.2	4.2

Our African subjects with diabetes did not use more hospitalizations or visits or pills than DMs in China or Europe or North America [22]–[26] but our subjects without diabetes used very few medicines and services relative to other countries. This underuse of medical care by the general population is a third driver of the large diabetes-associated differences that we report. Medical care in Africa is very costly relative to mean family income, difficult and time-consuming to access, and often unrewarding [27].

The present study has features and limitations that should be kept in mind when using and interpreting its results. First, as just described, our study samples excluded undiagnosed cases. Therefore, our reported differences and ratios probably overestimate medical services utilization per person among persons with undiagnosed diabetes. Second, as it is neither possible nor desirable to assign diabetes experimentally, our research design was observational. However, our case-control design is now the most widely used and accepted approach for measuring the economic and social impacts of diabetes, [22]–[26] for two reasons: (a) the effects and complications of diabetes are so numerous and serious that it is valid to assume that diabetes is the cause of the great majority of the events that are observationally associated with it, once the strong effects of age, sex, and socio-economic status (including three indicators: income, occupation and education) are controlled; and (b) diabetes complications are often not recognized as related to diabetes by caregivers and patients, which rules out bottom-up, medical record- or patient-based counting designs. [24] Our matching procedure appeared to succeed very well; not only were age and sex very similar across our DM and MCs, as would be expected, but DMs and MCs were also similar on family income per person and educational attainment, variables on which we did not match directly.

The communities we sampled were not entirely representative of their countries. In Mali, all the known persons with diabetes were randomly or exhaustively sampled in the three largest cities, but rural residents were excluded. In Cameroon, the sample largely consisted of consecutive users of the outpatient clinics of the university hospital in the capital city, a procedure that probably over-selected DMs who were more ill and more likely to use–and more likely to be able to afford–services. The Tanzanian sample was representative of all diagnosed residents of Temeke, a major suburban area of the capital province, but excluded both downtown Dar es Salaam and the interior of Tanzania. Compared to World Bank estimates of mean national income per capita for 2008, [36] when most of our interviews were conducted, mean incomes per family member among our DMs and MCs, respectively, were 3.4 and 2.6 times higher than the national average in Cameroon, 5.6 and 5.0 times higher in Mali, and 5.1 and 4.5 times higher in Tanzania. In South Africa, our sample contained almost exclusively black residents of a former segregated black township. Blacks comprise 79% of the South African population, [37] so our sample resembles the majority of South Africans racially. However, mean income per family member in our diabetes sample was still about 2.1 times higher than mean national income for black South Africans [38].

Fortunately, these large differences in relative affluence do not necessarily imply that our estimate of the absolute and relative impact of diabetes misrepresent Africans as a whole. Although urban Africans earn more income than rural Africans, they are also two to four times as likely to develop diabetes, [39]–[42] and higher-income residents of LMICs are two to three times more likely to develop diabetes, regardless of where they live. [43] Therefore, had we had the resources to undertake a fully population-based probablility sampling of Africans with diabetes, the resulting study subjects with diabetes would still have been predominantly urban and higher-income and–equally importantly–the control subjects without diabetes would have been much more rural and lower-income. As a consequence, we would have calculated much larger differences in medical care use than we have reported, because our matched control subjects undoubtedly used more medical services than similarly aged men and women in rural areas.

A final consideration in interpreting our results is that all our utilization data were obtained from patients’ self-reports. An extensive literature, albeit from industrialized countries, suggests that patients can recall the occurrence and many details of medical care encounters for up to three months, and recall the occurrence if not the details of hospitalizations for at least one year. [44]–[48] To the extent that patient recall fails, it usually fails in the direction of under-reporting, especially of outpatient visits. [47], [48] Under-reporting also increases with the number of encounters during the recall period. In this study, we minimized under-reporting by limiting counts of both visits and hospitalizations to a 90-day window and asking about the details of visits and hospitalizations for only the most recent encounter within that window. Any under-reporting in our data should have the effect of downwardly biasing our estimates of the effects of diabetes on medical care use, because subjects with diabetes had so many more encounters than controls. Note that, for medication use, we did not rely on recall but instead asked subjects to physically show us the medicines they were currently taking.

In view of the remarkably and unexpectedly large absolute and relative magnitude of additional medical care use that we observed to be associated with a diabetes diagnosis, there can be no doubt that diabetes imposes a huge burden on families and medical care systems in most, if not all Sub-Saharan African countries. The best previous estimates of the expenditures for medical care caused by diabetes assume a DM/MC ratio of 2.0. [2] Our results show that expenditure ratios for Sub-Saharan African countries and perhaps other LMICs are actually much higher. African governments have a strong economic incentive to prevent expensive diabetic complications through earlier diagnosis and effective, low-cost preventive treatment.
